# Sediment deposition and coral smothering

**DOI:** 10.1371/journal.pone.0216248

**Published:** 2019-06-19

**Authors:** Ross Jones, Rebecca Fisher, Pia Bessell-Browne

**Affiliations:** 1 Australian Institute of Marine Science (AIMS), Perth, Western Australia, Australia; 2 Western Australian Marine Science Institution (WAMSI), Perth, Western Australia, Australia; 3 The Oceans Institute and The Centre for Microscopy, Characterisation and Analysis, The University of Western Australia (UWA), Perth, Western Australia, Australia; University of Waikato, NEW ZEALAND

## Abstract

Dredging in the marine environment to create and maintain safe, navigable shipping channels, and subsequent disposal of the material at sea in dredge material placement sites (spoil grounds) can generate large quantities of suspended sediment that can impact upon epibenthic marine communities. For sensitive taxa such as hard corals, understanding the mechanisms of mortality and the spatial scale over which these occur is critically important for impact prediction purposes, management of dredging using zonation schemes, and also public perception. We describe the sediment deposition field from suspended sediment falling back out of suspension created around a large (7.6 Mm^3^) 1.5-year capital dredging project on a reef, using data from 2 weekly repeat observations of >500 individually tagged corals at multiple locations from 0.2–25 km from the dredging. The observations were supported by concurrent *in situ* measurements of proxy suspended sediment concentrations, underwater light, and sediment deposition (using optical backscatter sensors), and before and after surveys of seabed particle size distributions (PSDs). The distance at which 90% of the effect (from maximum to minimum) had dissipated (ED_10_) was 20 km away from the dredging for suspended sediment concentrations (estimated via nephelometry), and underwater light (measured using PAR sensors) associated with turbid plumes, 14 km for sediment deposition (measured using optical backscatter sensors) and 4.6 km for changes seabed clay and silt content (PSD analysis). The ED_10_ for smothering of corals (the build-up of pools of loose sediment on the surface that could not be removed by self-cleaning) occurred much closer still at 3–3.3 km or (0.5–0.6 km for an ED_50_). Smothering was common on encrusting and foliose forms where sediments accumulated in hollows and massive hemispherical forms where surface undulations (bumps) allowed sediments to pool. Smothering was never observed on branching species, even under extreme levels of sedimentation. Sediment smothering resulted in tissue bleaching and partial mortality (lesion formation), but if sediments were removed (by currents) bleached areas regained pigmentation over weeks and there was regrowth/reparation of lesions over weeks and months even before the dredging was completed. Overall sedimentation tolerance was highly related to coral morphology and surface inclination and the ability to avoid smothering by having uninterrupted downhill pathways for sediment transport across the colony.

## Introduction

Dredging in the marine environment to create and maintain navigable shipping channels and allow safe ship access, is a usual component of many large marine infrastructure developments [1]. Dredging involves the removal of sediment and/or rock from the seabed [2] and the excavation and subsequent disposal at sea in dredge material placements sites (spoil grounds) can generate large quantities of suspended sediment that can impact upon epibenthic marine communities [1, 3–5].

Well recognized cause-effect pathways include suspended sediment interfering with filtering and feeding mechanisms, increased turbidity (water cloudiness) changing light quantity and quality (for benthic primary producers), and increased sediment deposition causing smothering [5]. Predicting what may occur before a dredging program at the environmental impact assessment (EIA) stage, and managing dredging when underway through environmental monitoring, is predicated upon establishing a relationship between these dredging pressures (light reduction, suspended sediment, sediment deposition etc.) and biological responses in underlying communities i.e. developing thresholds or guidelines. In Australia, these are used in zonation schemes [6, 7] and proponents (i.e. those proposing to undertake the dredging) need to define, beforehand, zones of high and moderate impact, and also a zone of influence, which is where the plumes are likely to be seen but where there are no biological consequences. These predictions are important at the EIA stage of a project (where forecasts are made of possible environmental effects) and also in monitoring programs associated with the dredging phase, where proponents need to make sure they are compliant with the EIA predictions.

The problem is that the dredging pressures can act either individually or more likely in combination and also at different temporal and spatial scales. Knowing which is the most relevant is challenging especially because different communities (seagrasses, sponges and filter feeders, fish and corals) may each respond differently to different pressures [3, 8–10]. Managing dredging around scleractinian corals is difficult. They are phototrophic because of a mutualistic symbiosis with endosymbiotic dinoflagellates and hence are sensitive to light reduction in the water column. They can also be very sensitive to sediment deposition and have a number of different self-cleaning mechanisms to prevent sediment accumulation on their surfaces (reviewed by [11]). The process primarily involves muco-ciliary transport and corals continually shift sediments across their surfaces to prevent smothering–defined as the build-up of patches or pools of sediment that cannot be moved by self-cleaning. Smothering will reduce light availability under the sediment layer and can reduce gas (solute) exchange [12] and result in hypoxia/anoxia, and can ultimately lead to tissue necrosis (lesion formation) if the sediments remain in place and are not resuspended by waves and/or currents.

Establishing an evidence-based footprint of the scale of potential impacts associated with sediment smothering is important for dredging management. It is also important for perception of potential environmental effects associated with dredging [13]. While some turbid plumes generated by dredging can be seen for tens of kilometres [14, 15], the size of sediment deposition zones resulting in smothering of corals could be much closer to the source. Observations of corals during dredging could provide insights into these issues. Such an analysis was made possible from a major capital dredging project that occurred on the reefs in Western Australia. Since the dredging was conducted close to a diverse coral reef community there was an unprecedented level of monitoring to ensure any environmental effects complied with those approved by state and federal legislation. Over 500 individual coral heads from across multiple locations from hundreds of metres to tens of kilometres away from the dredging activity were photographed at frequent intervals for the extended duration of the project. At each site *in situ* water quality measurements of turbidity and underwater light levels were also made before and during the dredging. Sediment deposition is particularly challenging to manage at ecologically relevant scales i.e. mg cm^-2^ day^-1^ [5, 16–19], and in the project deposition was estimated using optical back scatter techniques [17, 20, 21], in some of the first measurements of their kind for a large dredging project. Some problems were encountered with the deposition sensors, which have since been modified and redesigned as described in [22]. Although deposition could not be measured in absolute terms (as mg cm^-2^ day^-1^), relative levels of deposition with distance from dredging could be calculated.

The images of the corals throughout the dredging program and the water quality datasets were made available by the dredging proponent for further study and analysis. Temporal and spatial patterns in turbidity and light levels during this and other capital dredging projects have been discussed in detail in [23, 24]. Patterns of coral mortality and dose response relationships have also been derived for the coral communities, with a focus on the uncertainty when combinations of stressors are involved[25]. The focus in this study is the settling of sediment and smothering of corals, the significance of sediment deposition as a cause-effect pathway and the size of the area where sediment smothering occurs compared to the long-distance movement of sediments in plumes.

## Materials and methods

### Water quality monitoring

Permission to undertake the dredging and to install water quality and coral health monitoring sites was given under ministerial approval statement No. 800, searchable on the Western Australia Environment Protection Authority (WA EPA) website: http://www.epa.wa.gov.au/all-ministerial-statements.

**Fig 1:**
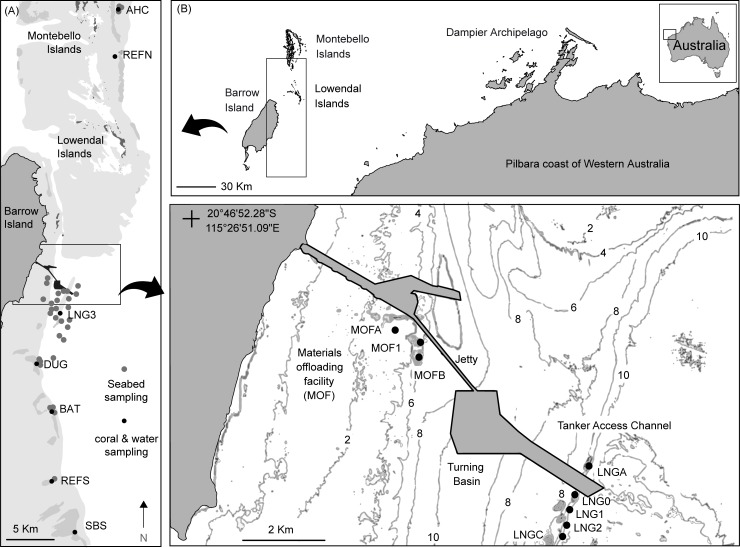
Location map. Location of Barrow Island in NW of Western Australia off the Pilbara coast, and the 15 water quality and coral health monitoring sites relative to the primary excavation areas (a materials offloading facility (MOF) sites and turning basin, and tanker access channel (LNG sites)).

From 19 May 2010 to 31 Oct 2011 (530 days) a large-scale capital dredging project was undertaken on the reefs around Barrow Island located ~50 km off the Pilbara region of NW Western Australia (Fig 1). The dredging occurred on a 7 days × 24 h basis, with scheduled stops only for maintenance and bunkering requirements. A combination of trailing suction hopper, cutter suction and back hoe dredgers, and bed levellers were used, with dredge material placement at an offshore disposal site. The material dredged was predominantly unconsolidated, undisturbed carbonate sediments forming a thin veneer (0.5–3 m deep) overlying limestone pavements, ranging from rubble to typically gravelly sand mixed with fine silts and clays [26]. A full description of the dredging program (type and nature of the dredging and substrates) as well as data collection and methods and analyses of the water quality monitoring data can be found in [23–25].

Before the start of dredging ~25 water quality and coral health (see below) monitoring sites were installed from a few hundred metres to several tens of kilometres away from the future dredging activities (Fig 1). Sites were installed June 2009 onwards and the installations were complete by the start of the dredging on May 20, 2010. During dredging there was a near continuous unidirectional, southerly movement of the sediment plumes throughout the 1.5 y study and for the purposes of this study, analysis of water quality and coral health was restricted to 15 sites to the south of the main dredging and 2 northerly reference sites located >25 km from the excavation area (Fig 1).

Photosynthetically active radiation (PAR) and turbidity, as nephelometric turbidity units (NTU), was recorded using instruments on seabed mounted frames[23, 24]. Light data were modelled to determine the sum of the per second quantum flux measurements and the daily light integral (DLI) calculated as mol photons m^-2^ d^-1^.

The instrument platform also included a sediment accumulation sensor which is based on optical backscatter sensor principle [17]. The sensor uses a light emitting diode (LED) source and fibre optic bundle set into a flat horizontal glass measuring surface. Sediment accumulating on the surface results in backscattering of light from the LED into the sensor. Every few hours a wiper removes accumulated sediment, resetting the sensor reading to zero and ideally creating a ‘sawtooth’ pattern in the sensor data, from which it is theoretically possible to calculate a sediment deposition rate [for a further explantion see 17, 20, 22]. In this study a sawtooth pattern was only occasionally observed and much more common were periods when sediment had obviously accumulated on the flat sensor but moved off and either resettled again or advected away from the sensor within the 2 h accumulation period. This prevented calculation of a sediment accumulation rate, but nevertheless provided information that measurable levels of sediment settling was occurring. The sensor output was averaged over a day and normalized to a scale of 0–1 for each site to produce a unit-less sediment deposition index (see [25]).

The particle size of surficial sediment samples was collected before dredging (September 2008–April 2009) and at 3 separate occasions after dredging approximately 1 year apart. Multiple scrapes of the surface 5 cm of sediment were collected by SCUBA divers within a 4 m^2^ area using pre-cleaned and labelled 250 mL plastic jars. Sediments were analysed by a commercial laboratory using wet sieving techniques for samples >500 μm and laser diffraction (Malvern Instruments Mastersizer MS2000) for fractions between 2 and 500 μm (ISO 13320–1).

### Coral health monitoring

The waters around Barrow Island support a diverse assemblage of tropical and subtropical marine fauna with over 250 species of hard corals ([27–29]). Species diversity is generally higher on the clearer water on the west coast of the island than the slightly more turbid, and lower energy waters along the eastern edge. Where the dredging took place, numerous scattered, isolated patch reefs support a varied mixed coral assemblage dominated by *Goniastrea* spp., *Porites* spp., *Euphyllia* spp., *Lobophyllia* spp., *Plesiastrea* spp., *Favia* spp., *Favites* spp., *Platygyra* spp. and *Acanthastrea* spp. and *Turbinaria* spp. and scattered hard corals such as *Acropora* spp. ([26]).

For each of the water quality monitoring sites a minimum of 50 coral colonies that were considered representative of the local reef community were tagged (marked with a unique identifier to aid future location). Additional colonies of massive Poritidae were also tagged to ensure a minimum of 20 colonies per site. Massive *Porites* spp. are difficult to identify underwater due to their small and variable corallites [30]. The species most likely included a mix of *P*. *lutea* and *P*. *lobata*, which are the most common massive *Porites* spp. in the region [28].

The colonies were photographed every ~14 d for the 530 day duration of the dredging, until 11 November 2011, and for most sites 27−40 surveys were undertaken over the dredging period. From the photographs the morphology of each colony was recorded as either encrusting, foliose, corymbose, branching or massive. Each tagged colony photograph was then scored according to the proportions of cover by sediment and for the *Porites* spp. covering by mucous sheets[31], using a categorical scale ranging from 1 to 7, where 1 = 0%, 2 = 1−5%, 3 = 6−33%, 4 = 34−65%, 5 = 66−95%, 6 = 96−99%, and 7 = 100% coverage. Where it was clear from the photographic time series that a colony had been moved or dislodged either by SCUBA divers during the photographing process or swells and waves associated with tropical storms and cyclones Bianca or Carlos, the corals were excluded from all further analyses.

Colonies often became partially covered in sediment (see below) and because of the photographic time sequence it was possible to follow the fate of the underlying tissue in time. If sediments were ultimately washed off the coral surface by waves or currents revealing live tissue, the sediment covered tissues was classified as ‘live’ through the photograph sequence. If the sediments remained on the surface, or once washed off revealed a dead surface, the tissue was classified as ‘dead’ from when the sediment smothering was first observed. The same principle was used in the case of mucous sheet formation in massive *Porites* spp. ([31]). The number of observations of sediment cover and mucous sheet formation (*Porites* spp. only) were determined and summarised for each site.

### Statistical analyses

Light, suspended sediment concentration (SSC) and deposition at different distances from the dredging activities was summarised as fortnightly maximum 14 d running mean DLI, 14 d running mean nephelometrically-derived SSC (using a nephelometric turbidity unit (NTU) to SSC conversion factor of 1.8 (see [23, 24]), and 60 d running mean sediment deposition index. Running mean time scales were selected as those best predicting coral mortality [25].

Distance decay relationships were fitted for these water quality parameters using mixed model regression, with distance from dredging activity as the predictor, and fortnight number and site code included as random effects. Distance was modelled on a log scale, as previous analysis had found an exponential decay in water quality conditions with distance from dredging. Appropriate transformations were applied to linearize the relationship with log_10_(distance) and to ensure normality. This included modelling the log_10_ of NTU, the square-root of DLI and the logit of sediment deposition, which was an index between 0 and 1.

Sediment particle size distribution was expressed as relative percentage of particle sizes in each of the four classes gravel (>10–2 mm), sand (2000–62.5 μm), silt (62.5–4 μm), and clay (<4 μm), and a distance decay relationship fitted using the logit of the proportion in the combined silt and clay size class against log_10_ distance as above.

The percentage of colonies having a mucous covering or sediment smothering score of 3 or greater i.e. >5% mucous or sediment cover was also modelled on a logit scale as a function of log_10_ distance, as was the probability of non-zero mortality of *Porites* spp. (as reported in[25]).

From the distance decay relationships effect distances were calculated 50% (ED_50_) and 10% (ED_10_) from the predicted value at the farther site distance (34.8 km) to that of the predicted value at the closest site distance (190 m). All relationships were fit as Bayesian models using Stan and the rstanarm package package in R [32] with uninformative priors. Estimated 95% credible bands were calculated from five Markov chain Monte Carlo chains (10,000 burn-in, 20,000 iterations).

## Results

### Observations from the coral colony monitoring program

**Fig 2:**
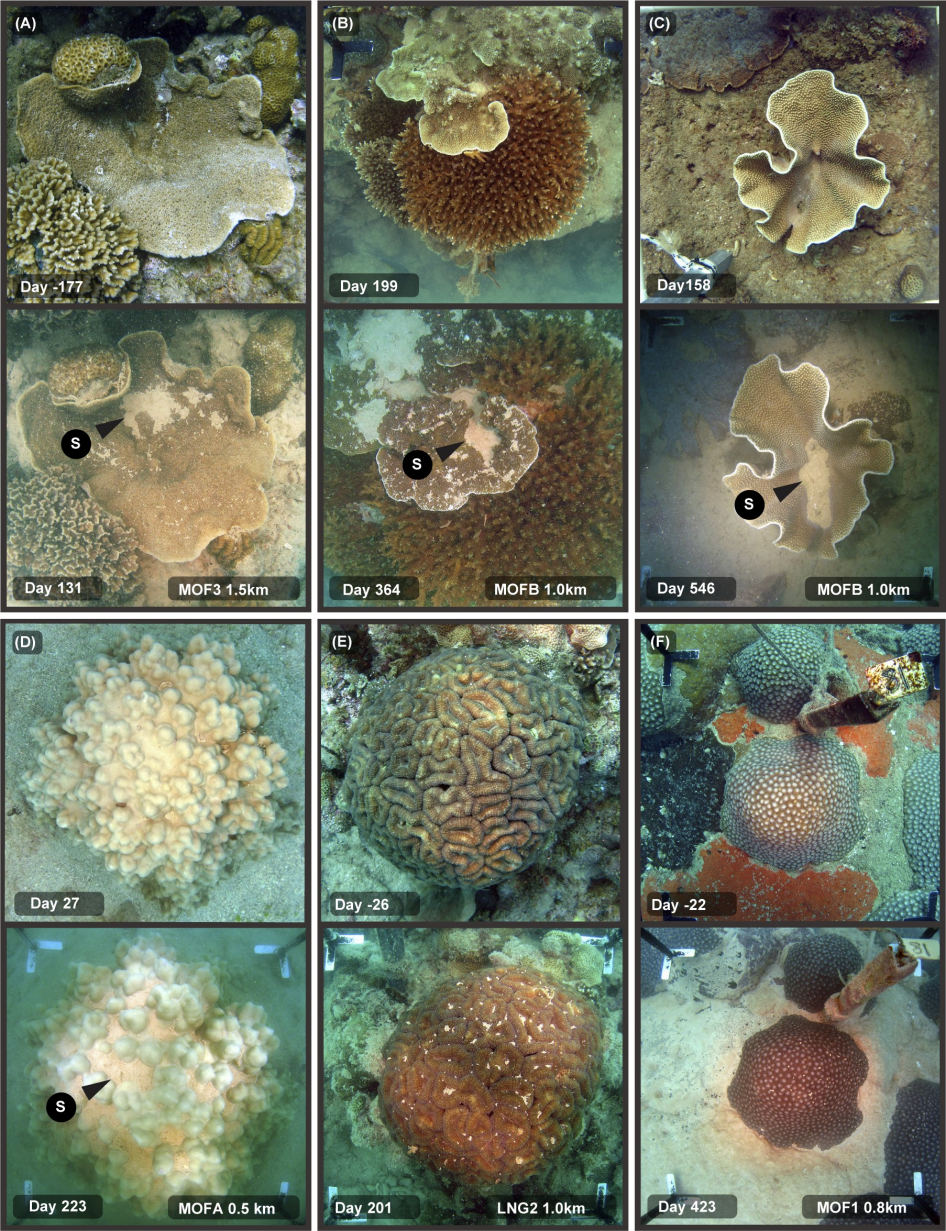
Sediment smothering in different coral morphologies during a large-scale capital dredging project–see text for explanation. Legends on the images indicate the site name (see Fig 1), distance from dredging activities (km), and days since the start of dredging (19 May 2010). The images show the differing susceptibility of a range of morphologies to sediment deposition with colonies with concave areas and depressions on the surface more commonly accumulating sediment compared to more hemispherical, rounded morphologies. For the arrows, S = sediment accumulation.

There was clear evidence of sediment smothering associated with the dredging program at sites very close to the dredging activities, with patches of loose, unconsolidated, fine sediment collecting on the surfaces of corals near the dredging activities. This sediment ‘smothering’ was most commonly observed in flattened species such as *Montipora* and *Podabacia* spp. corals (Fig 2A and 2B) and in foliose *Turbinaria* spp. corals (Fig 2C). Sediments typically became trapped in concave depressions or ‘hollows’ on the surfaces, sometimes building up deposits several millimetres thick. Sediment build up was also frequently observed on the uppermost section of some massive *Porites* spp., especially those with more rugose, bumpy surface morphologies where sediments accumulated in valleys between neighbouring bumps or protrusions (Fig 2D, see below). Occasionally small (mm sized) patches of sediments were observed on smooth hemispherical or rounded massive colonies such as *Lobophyllia* spp. and *Diploastrea* spp. (see Fig 2E and 2F); however, these morphologies were typically very capable of rejecting sediments even during periods of extreme levels of deposition resulting in smothering of surrounding surfaces (see background of Fig 2F).

**Fig 3:**
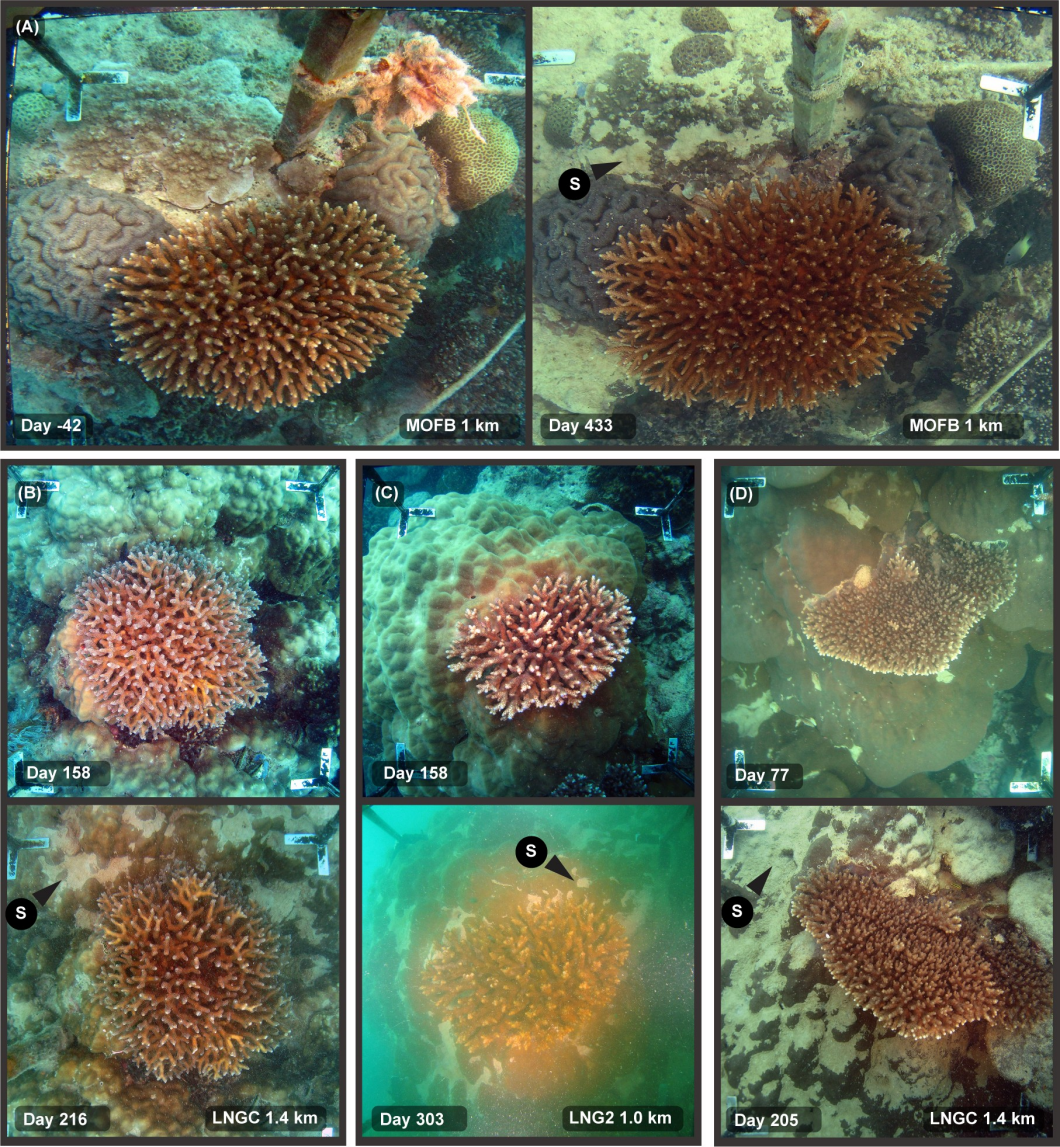
Sediment smothering during a dredging project, showing different susceptibility of different morphologies–see text for explanation. Legends on the images indicate the site name (see Fig 1), distance from dredging activities (km), and days since the start of dredging (19 May 2010). (A) A mix of morphologies including massive (hemispherical) colonies, encrusting and branching (tabulate) morphologies showing smothering of the flat, low lying encrusting form at Day 433. (B–D) Images showing the sediment clearing abilities of branching morphology compared to underlying massive, hemispherical morphology of the *Porites* spp. For the arrows, M = mucus or mucous sheet, B = bleaching, S = sediment accumulation, R = reparation/repair.

**Fig 4:**
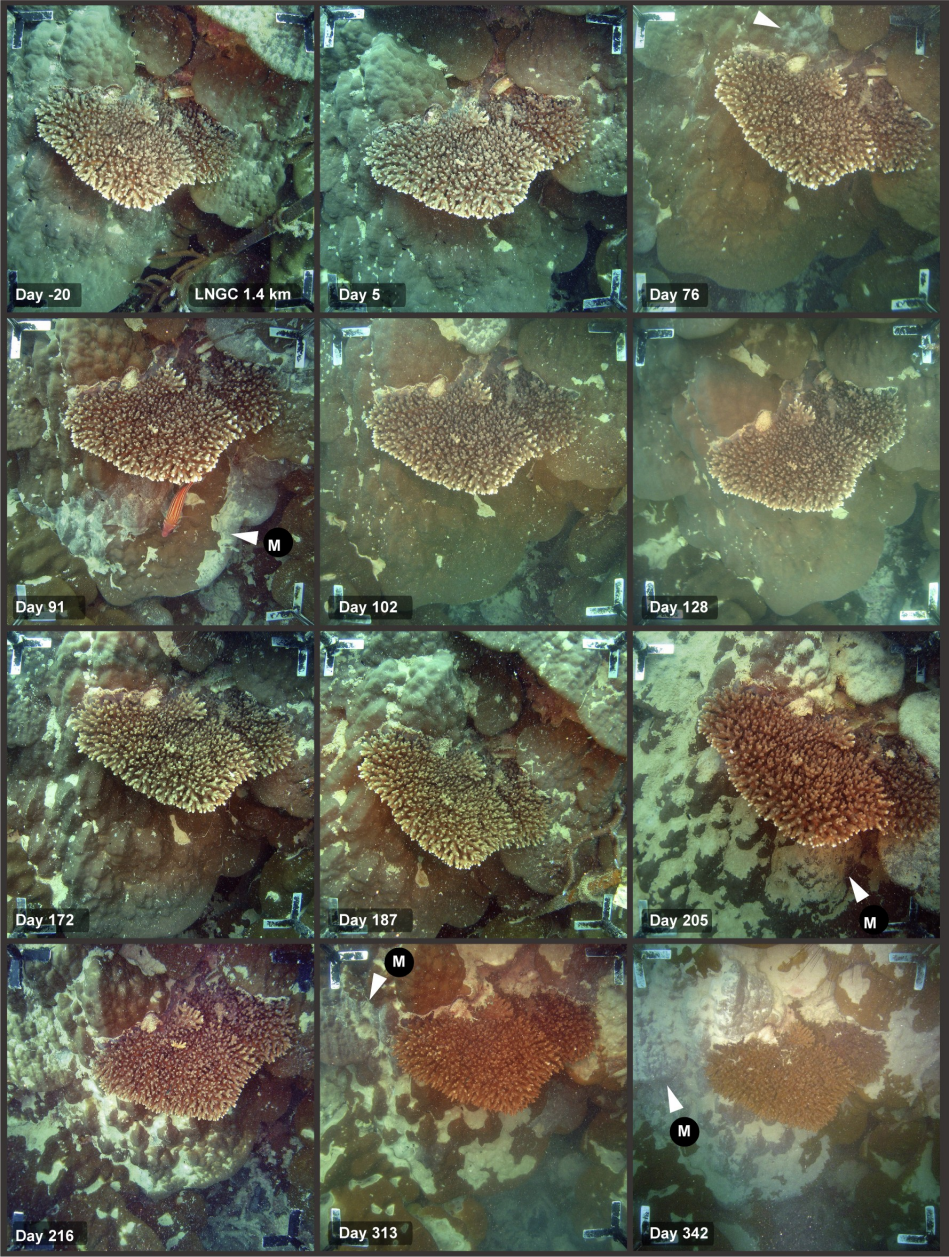
Time series of images showing sediment smothering during a dredging project, showing the different susceptibility of two different morphologies–see text for explanation. Legends on the images indicate the site name (see Fig 1), distance from dredging activities (km), and days from the start of dredging (19 May 2010). The images show the superior sediment clearing abilities of branching (tabulate) morphology of the *Acropora* spp. compared to underlying massive, hemispherical morphology of the *Porites* spp. which becomes smothered with fine sediments. White arrows indicate mucous sheet formation (see text).

The relative sediment clearing abilities across a range of morphologies is also indicated in Fig 3A, showing a cluster of branching, hemispherical and encrusting colony morphologies before and then near the end of the dredging program. Extensive smothering of the low relief, encrusting *Montipora* spp. has occurred whilst the overlying tabulate *Acropora* spp. and neighbouring hemispherical *Lobophyllia* and *Diploastrea* spp. show little or no evidence of smothering. Fig 3B–3D show branching *Pocillopora* and *Acropora* spp. colonies overlying massive, hemispherical *Porites* spp. colonies showing smothering of the *Porites* spp. but no accumulation of sediment on the branching forms. Fig 4 shows an extended ~360 d sequence of images of the two colonies in Fig 3C, showing no evidence of sediment accumulation on the tabulate *Acropora* spp. despite near complete smothering of the underlying *Porites* spp.

**Fig 5:**
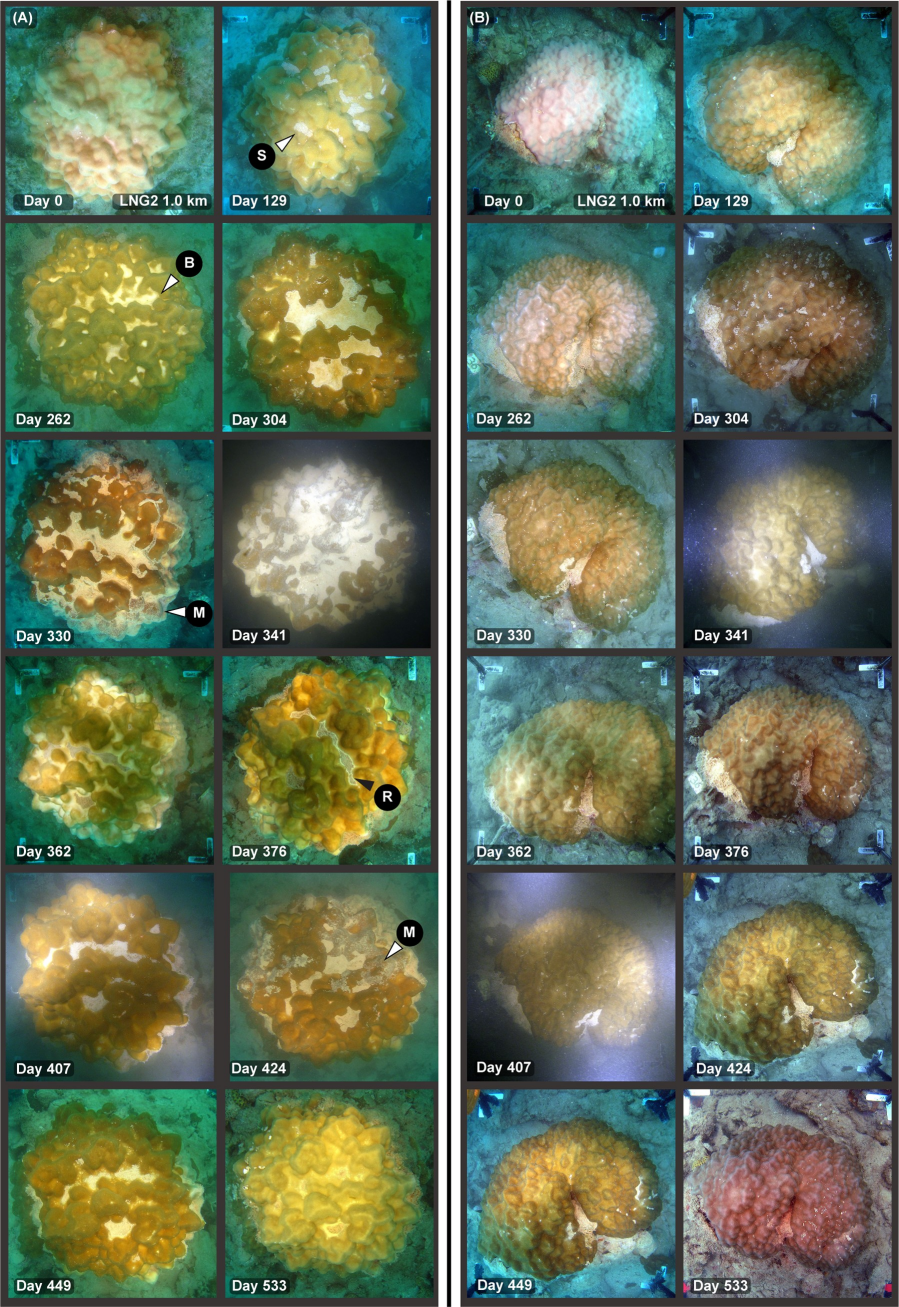
Time series of images showing sediment smothering during a dredging project, showing the different susceptibility of different morphologies in *Porites* spp.–see text for explanation. Colonies in (A) and (B) were from site LNG2, 1.0 km away from the dredging. Legends on the images indicate days since the start of dredging on 19 May 2010 (see text). The images show the contrasting the sediment clearing capacity of the *Porites* spp. colonies with subtly differing morphologies (bumpy versus smooth, see text). For the arrows, M = mucus or mucous sheet, B = bleaching, S = sediment accumulation, R = reparation/repair contrasting.

Massive, hemispherical *Porites* spp. with smoother surfaces were more efficient at sediment rejection than more rugose, bumpy forms and Fig 5 contrast the fate of two neighbouring *Porites* spp. over the 530 d of the dredging. Surface smothering was regularly observed in the bumpy form were sediments became trapped in valleys as opposed to the smoother form (cf day 129, 304, 341, 407 in Fig 5A and 5B).

**Fig 6:**
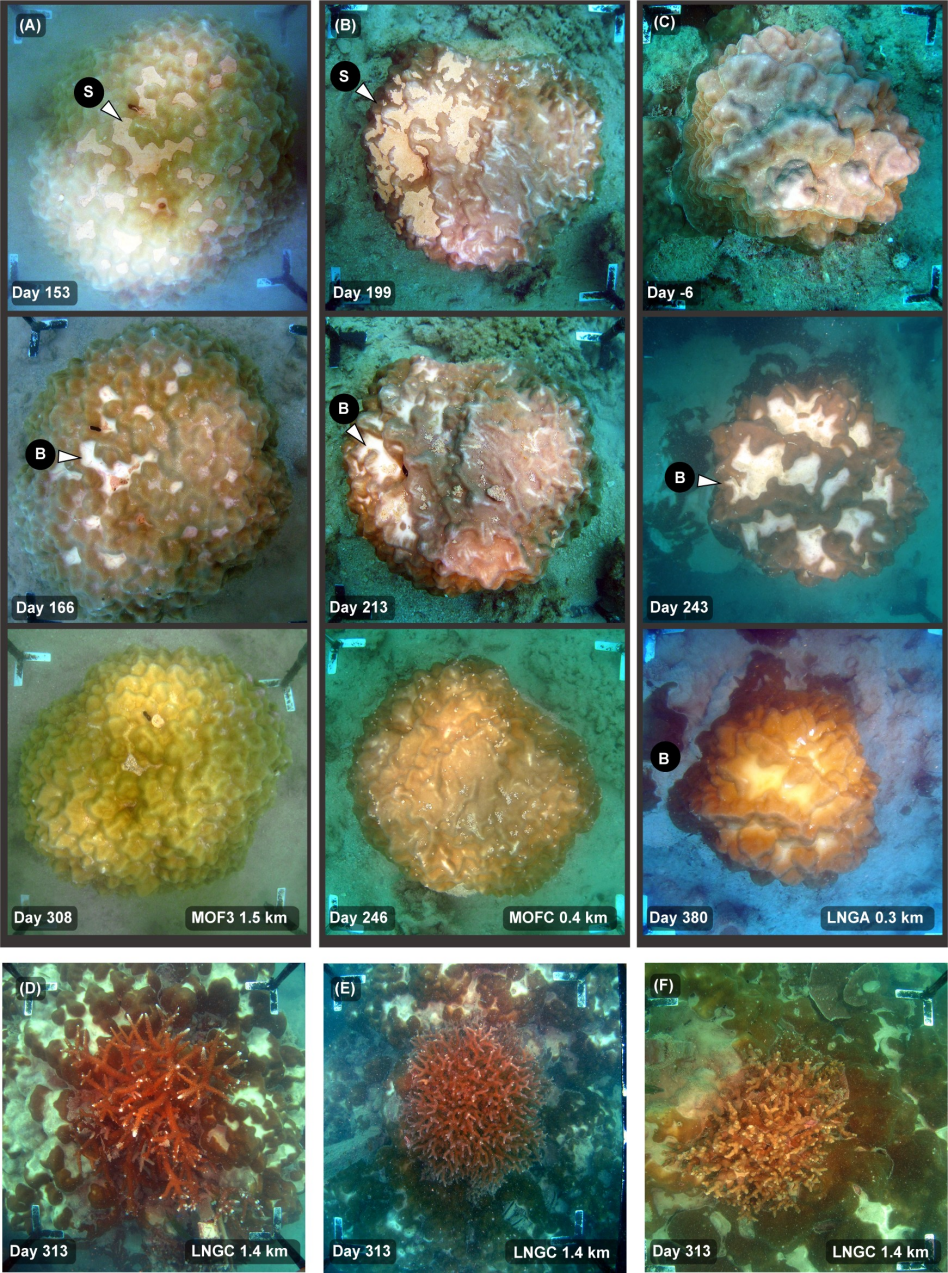
Effects of long-term sediment smothering on corals during a dredging project–see text for explanation. Legends on the images indicate the site name (see Fig 1), distance from dredging activities (km), and days since the start of dredging on 19 May 2010). The time series for colonies (from three different sites) over 380 days of the dredging show tissue bleaching in surface hollows of massive *Porites* spp. that were at some stage covered with sediment and eventual recovery of pigmentation. (D-F) Show similar sediment smothering and/or bleaching in hollows of the massive *Porites* spp. (background) whilst the overlying branching *Acropora*, *Pocillopora* and *Porites* species in the foreground show no smothering. For the arrows, M = mucus or mucous sheet, B = bleaching, S = sediment accumulation, R = reparation/repair.

Using the time series of photographs, it was possible to follow the fate of colonies once smothered. If sediments that had accumulated in hollows on the surface was re-suspended from the colony (by waves and/or currents), the tissues underneath was often discoloured or ‘bleached’ (see Fig 6A–6I). Bleached areas often regained their pigmentation (see Fig 6G, 6H and 6I). Fig 6J–6I shows normally-pigmented branching *Acropora*, *Pocillopora* spp. colonies overlying massive *Porites* spp. colonies where there is an unusual variegated appearance characterized by bleaching of the hollows and valleys between bumps. The pied appearance is interpreted as being caused by prior smothering by sediment based on the similarity to the pattering seen in Fig 6J–6I.

The longer time sequence in Fig 5A, also shows bleaching of areas (see arrow, day 262) where sediments have been re-suspended off the coral’s surface. This is likely to be have been caused by waves and swell associated with Tropical Cyclone Bianca, which passed by Barrow Island as a category 2/3 cyclone, 105 nm away from the island at its closest point, although no effects of cyclones Bianca or Carlos on hard coral cover on the reefs around Barrow Island was reported by [33]. More sediment was observed accumulated in the hollows on days 304, 330 and especially on day 341 where the coral’s surface is approximately 50% smothered by sediment (Fig 5). When next observed on day 362 the sediment had again been re-suspended from the surface showing more bleached areas (Fig 5). These bleached areas quite rapidly regained their pigmentation (over a period of 2 weeks) and our interpretation of the white leading edge of the coral tissue (see arrow in Fig 5A at Day 376) is that the coral is repairing or re growing over a central lesion in the deepest part of the hollow. After several more photographs showing sediment smothering in the hollows, the colony at the end of the dredging has only a few small patches of sediments which may be lying on a lesion or bleached tissue (see arrow in Fig 5A at day 533).

**Fig 7:**
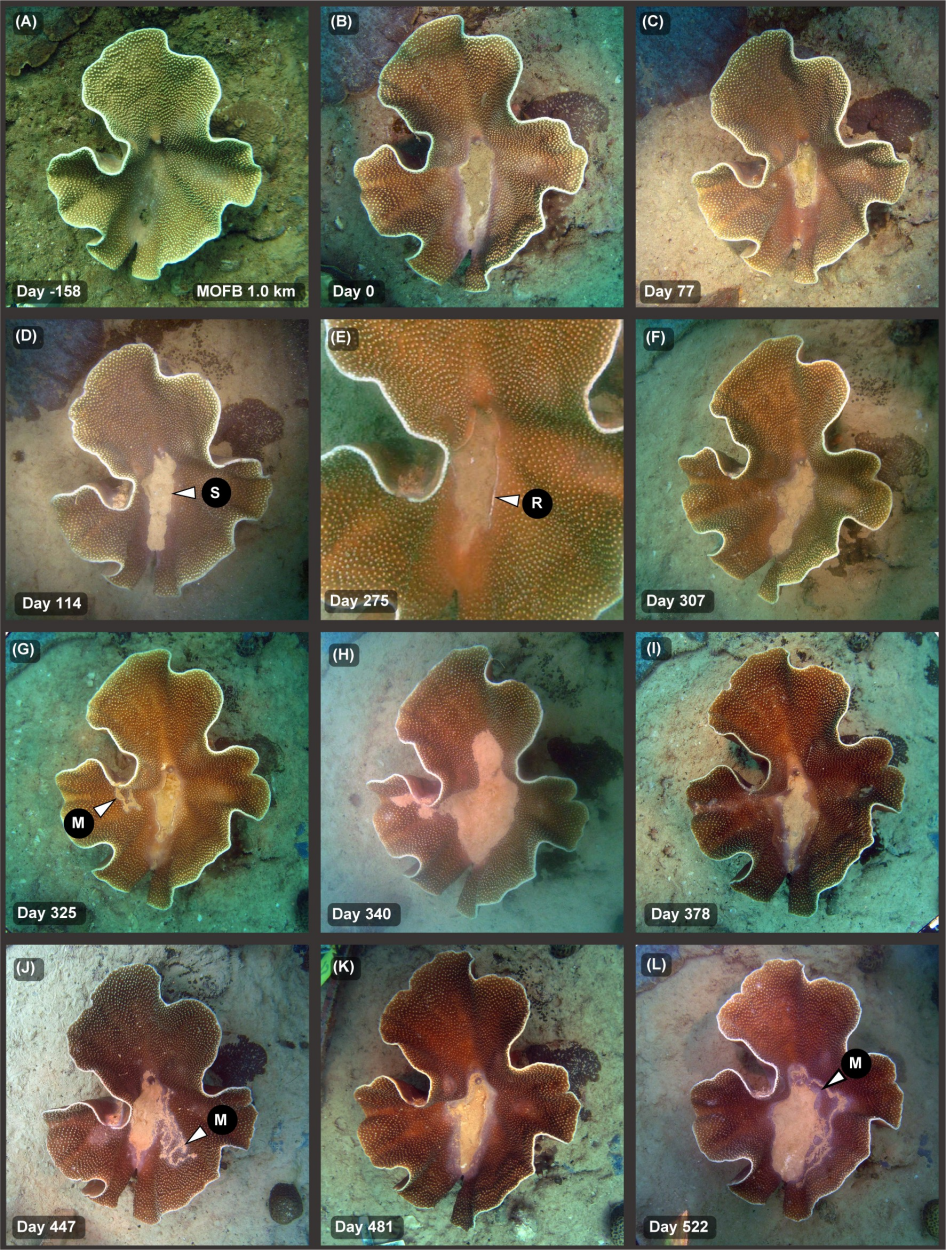
Time series of images of sediment smothering in a foliose *Turbinaria* spp. during a dredging project–see text for explanation. Shown is a coral colony located at site MOFB (see Fig 1) 1.0 km away from the nearest dredging over the 533 d Barrow Island capital dredging program, showing various degrees of smothering, bleaching and recovery/repair (see text). For the arrows, M = mucus or mucous sheet, B = bleaching, S = sediment accumulation, R = reparation/repair.

Similar patterns of smothering, resuspension, bleaching and recovery/repair were observed in *Turbinaria* spp. with an ‘open’ foliose morphology (Fig 7). In this colony a central lesion formed before the dredging started, and over the duration was seen with either a high sediment content filling the central lesion (Fig 7B, 7D, 7F, 7H, 7J and 7L) or with a low sediment content (Fig 7A, 7C, 7E, 7G, 7I and 7K). The photograph on day 275 (19 February 2010) was after Cyclone Bianca, and the central hollow was virtually sediment-free, with a white growing edge around a central lesion (see arrow Fig 7).

**Fig 8:**
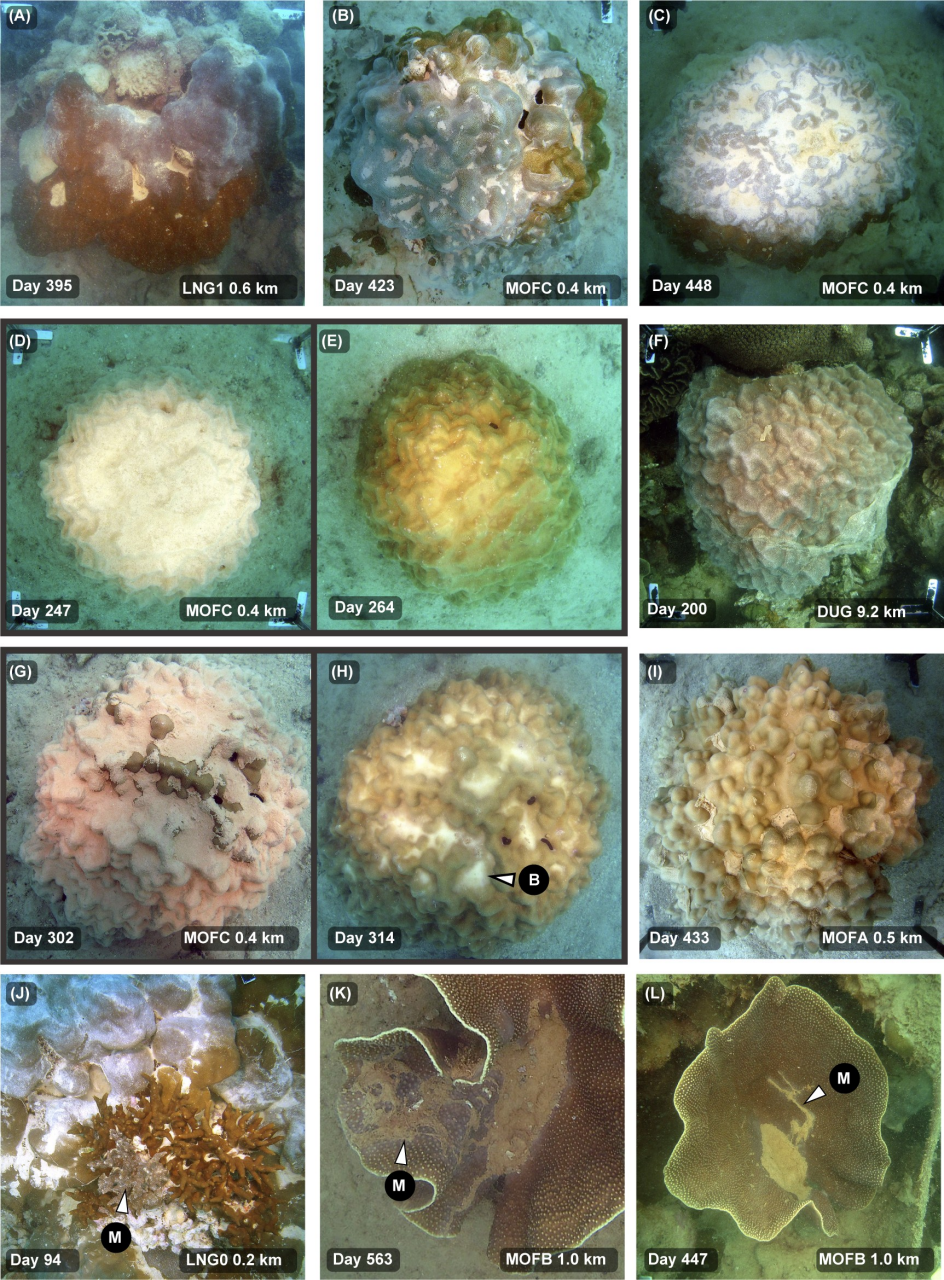
Mucous sheet formation and mucus production in massive, foliose and branching coral colonies during a dredging project–see text for explanation. Legends on the images indicate the site name (see Fig 1), distance from dredging (km), and days since the start of dredging (19 May 2010). For the arrows, M = mucus or mucous sheet, B = bleaching.

One of the most conspicuous physiological responses of the *Porites* spp. to the dredging activities was the production of mucous sheets (see [31]). These sheets, seen forming in Fig 8A, sometimes enveloped large parts or even the whole colony (Fig 8B–8D and 8G). Once formed (or forming) the sheets became fouled with sediments becoming progressively opaque. The mucous sheets eventually slough from the surface (Fig 8F and 8I) revealing clean, largely sediment-free surfaces underneath (Fig 8E, 8F, 8H and 8I). Bleaching was occasionally observed on the corals surface once the sheet had been sloughed (Fig 8H).

Some colonies were observed forming multiple sheets over the dredging project (see white arrows in Fig 4, Fig 5, and Fig 3 of [31]. Mucous sheet formation was also occasionally seen on branching *Porites* spp. (see Fig 8J). Mucus production was observed in some foliose *Turbinaria* spp. but presented as strands or web-like meshes that gathered sediments and were presumably sloughed or gathered in the central hollow (see Fig 7G, 7J and 7L and Fig 8H and 8I).

**Fig 9:**
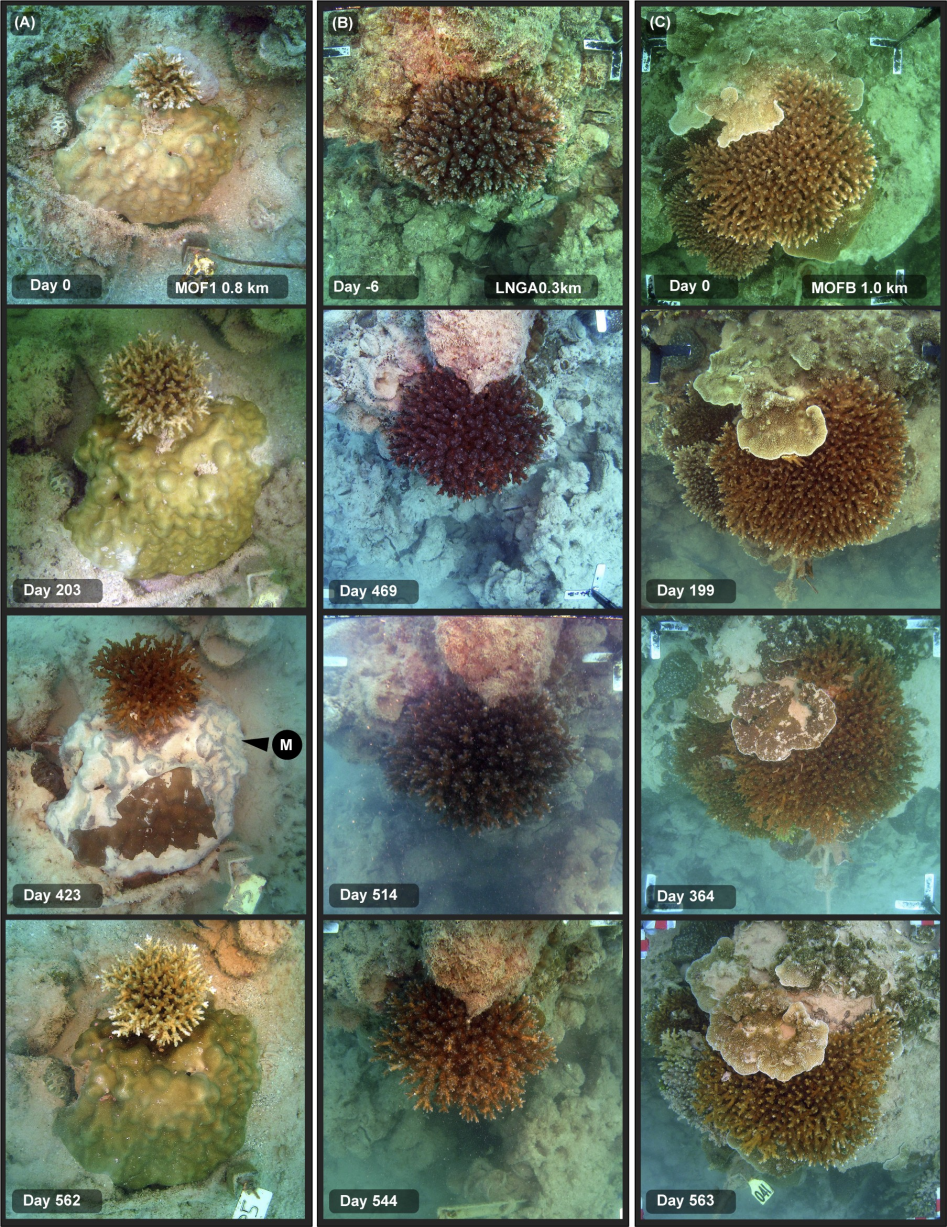
**A, B, C. Time sequence of branching, massive and encrusting coral colonies during a dredging project–see text for explanation**. Legends on the images indicate the site name (see Fig 1), distance from dredging activities (km), and days since the start of dredging on 19 May 2010). Images show the growth of some colonies over the dredging program. For the arrows, M = mucus or mucous sheet.

Fig 9 shows evidence of growth of branching and encrusting species during the dredging program.

### Water quality monitoring

**Fig 10:**
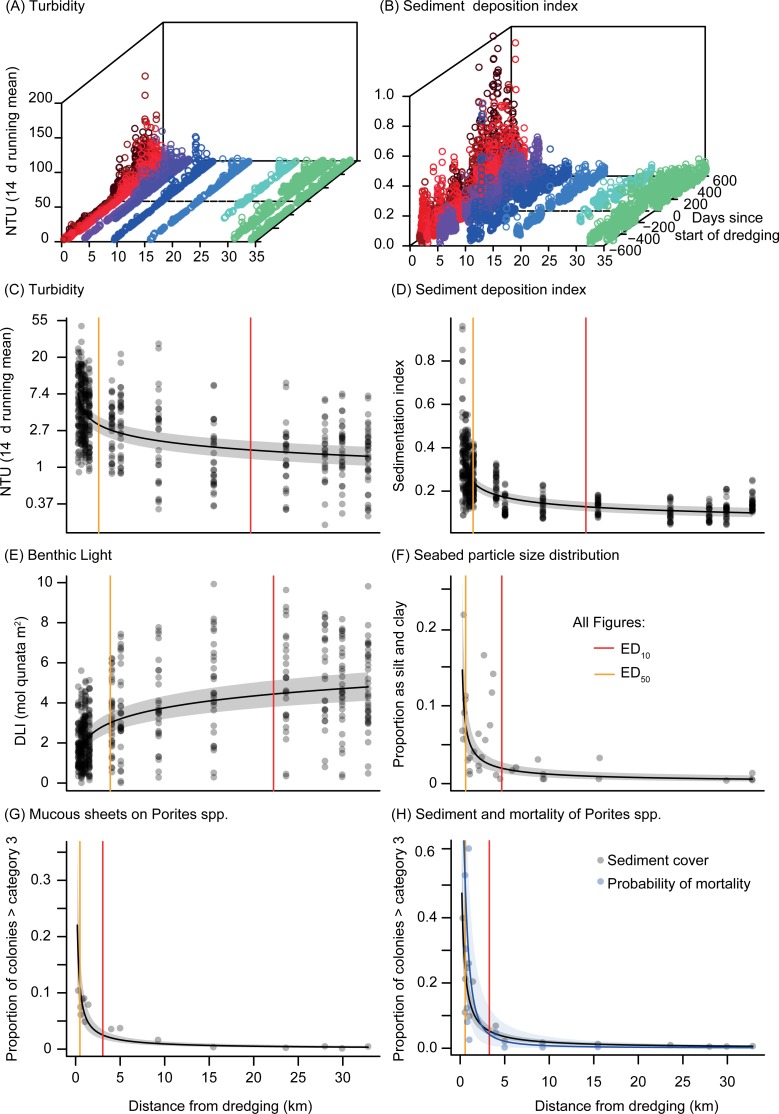
**A-D. Distance decay relationship for physical parameters (turbidity sedimentation, light availability) and biological parameters (mucus sheet production, smothering and mortality) during a large-scale dredging project.** Shown are (A) the average daily nephelometry-derived suspended sediment concentration (SSC) and (B) Sediment deposition index (range 0–1), against distance (km) from dredging (see Fig 1) during the pre-dredging, baseline phase, and from the start of dredging (Day 0, 19 May 2010) until completion (4 Nov 2011). The 2D plots show (C) fortnightly maximum 14 d running mean NTU, (D) 60 d running mean sediment deposition index, (E) 14 day running mean daily light integral over the dredging phase, the proportional increase in clay and silt in benthic sediments (F), the proportion of coral colonies having a mucous score of >3 (6–33% coverage) (G), the proportion of coral colonies having a sediment cover score of >3 (6–33% coverage) and estimated probability of non-zero *Porites* spp. mortality (H). Red and orange lines indicate the estimated distance of 10% (ED_10_) and 50% (ED_50_) effect as determined by fitted regression.

Spatial and temporal patterns in water quality (turbidity and underwater light) associated with the dredging have previously been described in detail[23, 24]. Briefly, nephelometrically-derived SSCs as well as sedimentation increased markedly from the start of the dredging campaign (at Day 0) compared to the baseline-pre-dredging phase (Fig 10A & 10B). There was a strong relationship with distance from the dredging activity for all three water quality parameters, allowing calculation of an effect distance (ED) (Fig 9). Estimated distance of 10% effect (ED_10_) for SSC (NTU) was at 20 Km (Fig 10C, Table 1). Associated with the high SSCs (hence turbidity), the ED_10_ for 14 day running mean light (as DLIs) was estimated at 22 km (Fig 10B and 10E, Table 1). Distance of observed sedimentation were slightly less than for turbidity and light, with estimated ED_10_ at around 14 km from the source of dredging (Table 1, Fig 10).

**Table 1:** Effect distances (ED) in km for a 90% decline (ED_10_) and 50% decline (ED_50_) in physical pressure parameter: SSCs (NTU), benthic light (DLI), sediment deposition and changes in silt and clay content and (B) biological response parameters including: sediment smothering and mucous sheet covering in massive *Porites* spp. The physical and biological parameters were measured at 17 locations at different distances from the dredging activities (Fig 1). Effect distances were calculated from the predicted value at the farther site distance (34.8 km) to that of the predicted value at the closest site distance (190 m), with confidence bounds estimated from a posterior sample from Bayesian model fits.

	ED_10_ (km)	95% CI (km)	ED_50_ (km)	95% CI (km)
(A) Physical pressure parameters				
Nephelometry-derived SSCs	20	9.7–33	2.5	1.2–4.9
Daily Light Integrals	22	11–33	3.8	1.9–7.6
Sediment Deposition Index	14	8.3–28	1.4	0.83–2.2
Particle Size Distribution	4.6	2.7–8.6	0.56	0.19–1.0
(B) Biological response parameters				
Mucus cover >6−33% (Category 3)	3.0	2.0–4.5	0.49	0.21–0.81
Sediment cover >6−33% (Category 3)	3.3	2.4–4.3	0.56	0.34–0.81

**Fig 11:**
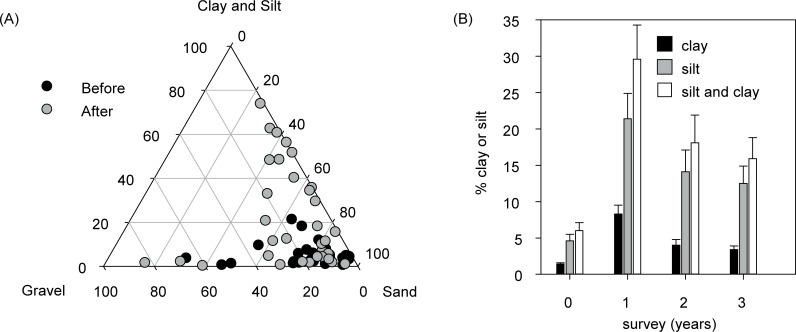
Changes in the particle size distribution of surficial seabed sediments collected from multiple locations from 200 m to 34 km away from the dredging before and after dredging. Shown are A) ternary plot of the percentage composition of the sediments collected (see Fig 1) expressed as percentage gravel, sand and silt and clay (62.5–0.02 μm). (B) Site level summaries of the clay and silt content immediately after dredging compared to before dredging, against distance (km) from the dredging.

Before the dredging the particle size distributions of surficial sediments collected from 200 m to 35 km away from the dredging were predominantly sand sized (Fig 11A). In the first survey after the dredging, there was a marked increase in the clay and silt (0.2–62.5 μm) composition, seen as an upward movement of the grey circles in Fig 11A. When expressed as a change from the baseline period, the combined clay and silt fraction showed a clear distance–decay relationship and the estimated distance of effect (ED_10_) was 4.6 km (Fig 9F). On a percentage composition basis, and for samples within 5 km from the dredging, the combined silt and clay content was 5 times higher after the dredging (Fig 11B), and although halving in subsequent surveys was still ~2.6 times the pre-dredging value 3 years after the dredging (Fig 11B).

### Patterns of sediment smothering and mucus sheet production in *Porites* spp.

Over the 553 d of dredging 660 individually tagged coral colonies were examined with 6,920 (i.e. colonies × time) observations of *Acropora* spp. and Pocilloporidae colonies (branching/corymbose morphologies), and 10,821 observations of massive *Porites* spp. colonies. As discussed previously, there were no examples of sediment smothering in the *Acropora* spp. and Pocilloporidae colonies (branching/corymbose morphologies, see also Fig 3).

The percentage of observations of massive *Porites* spp. corals with >5% mucous cover (category 3 and above) ranged from ~5–10% at the sites <1.5 km from the dredging to <2% at sites ≥~5 km away (Fig 10G). The estimated distance of 10% effect (ED_10_) for mucous sheet formation (6–33% of the colony surface) was at 3 km (range 2–4.5 km, Table 1). Patterns of sediment smothering on *Porites* spp. were very similar to those of mucous sheet formation, with the number of observations of corals with >5% mucous sheet cover (category 3 and above) ranging from ~10–40% at the sites <1.5 km from the dredging to <2% at sites >5 km away (Fig 10H). The estimated distance of 10% effect (ED_10_) for sediment smothering (6–33% of the colony surface) was only slightly greater than that observed for mucous sheet formation, at 3.3 km (range 2.4–4.3 km, Table 1) and showed a very similar pattern to that for estimated probabilities of non-zero mortality (Fig 10H).

## Discussion

Sediment deposition resulting in smothering of corals leading to mortality is a significant cause-effect pathway associated with dredging and turbidity generating activities near coral reefs. In comparing the spatial patterns of turbidity and light attenuation, seabed particle size distribution and sediment deposition, with biological responses including sediment smothering and mucous sheet formation in corals, the sediment deposition field was approximately an order of magnitude less than the distances travelled by the plumes. Coral mortality occurred within a few kilometres south of the dredging and within the area defined by the deposition field. Establishing evidence-based footprints of the scale of potential impacts associated with sediment smothering as compared to elevated turbidity and dredging related plumes is important for dredging management, and also for perception by the public and regulators of potential environmental effects [13]. Equating risk based solely on the extent of visible plumes could be very misleading as to the overall spatial effects of dredging projects.

### Spatial effects

A previous study of the turbid plumes associated with this dredging project showed they could be identified from satellite images up to 30 km away from site of excavation [34]. Analyses of water quality data showed elevated SSCs and reduced light availability (identified by a median value above the 80^th^ percentile of the baseline, pre-dredging data) and occurred 17–22 km from the site of dredging [23]. In this study, a different approach (the ED_10_) was used to contextualize the spatial effects for a range of sediment-related metrics. For turbidity and light the distance from dredging at which 90% of the effect–from maximum to minimum–had dissipated was in the order of 20 km, similar to the estimates of [23]. The ED_10_ from the sediment deposition sensors was closer at 14 km. Measurable changes in the seabed clay and silt content occurred much closer to dredging with an ED_10_ of 4.6 km. For biological effects such as smothering of corals and mucous sheet formation (see below) ED_10_ values were even closer to the dredging, at around 3–3.3 km. More substantial impacts, such as a 50% effect on coral colony parameters (ED_50_, Table 1), occurred still closer to the dredging activity, at >0.5–0.6 km.

The distance decay relationship for elevated SSCs and a reduction in benthic light [23] and sediment deposition shown in this study gives considerable authority to the use of zonation schemes (see [6, 7] as frameworks to manage dredging projects near sensitive marine environments. Clearly, spatial effects will be highly project specific and for contextual purposes this was a very large scale (7.6 Mm^3^), 1.5 year capital dredging project with marked effects on water quality. The local oceanography was also unusual and characterized by unidirectional southerly flow over the project duration [23, 34].

The sediment deposition hazard associated with dredging activities arises because sediments are mobilized from the seabed into a typically low energy water column, where turbulence and hydrodynamic forces are usually insufficient to keep them in suspension. This results in elevated rates of deposition and the creation of a deposition field that can lead to smothering of sensitive organism such as corals when the self-cleaning capabilities are exceeded [5]. In this study the resettlement of the suspended sediments resulted in a 5 times increase in the silt and clay content of the seabed after dredging. Such an increase has been reported previously in dredging projects [5], but in this study the particle size distribution analyses showed increased silt/clay was still 2.5 times higher than the pre-dredging level 3 years after the dredging activities were completed. Increased siltification and the potential for increased turbidity by wind and wave resuspension is a long-term environmental legacy of dredging projects and the ecological consequences to nearby benthic communities have yet to be properly understood.

The response of coral colonies to high sediment loads varied substantially across coral taxa, as well as across different growth forms even within taxa. Smothering was very morphology-specific and common on encrusting, foliose and certain massive morphologies, but was never observed on branching species *Acropora*, *Pocillopora* and *Porites* spp. species at any stage throughout the dredging program. In morphologies where smothering occurred, sediments were commonly seen accumulating in concave depressions or ‘hollows’ (*sensu* [35]) on the surface, as has been reported previously [11, 36, 37]. This was particularly evident for the massive *Porites* spp. colonies where sediments accumulated on more rugose, bumpy morphologies than those with smoother surfaces (cf Fig 5) as a result of sediments becoming trapped in local minima.

### Sediment clearance model for corals

Corals clear their surfaces using a range of different active (energy-requiring) mechanisms [11, 36, 38], of which the muco-ciliary transport process [35, 39] is the most important. Passive processes are also involved which are primarily associated with gravitational forces related to surface inclination and water movement [11, 38, 40, 41]. Some corals have been referred to as ‘passive shedders’ and using passive processes to remove sediments or using active or passive sediment removal mechanisms [4]. However, recently it was shown that accumulation rate of fine silt and clay was only marginally lower on dead branching skeletons of a range of morphologies than on flat, two dimensional coral surfaces [42]. The implication is that sediments are not being passively shed and it seems unlikely that passive process alone can clear sediment from corals as sediments also become attached or ‘embedded’ [39] in the surface mucus layer of corals on initial contact. Perhaps passive shedding of sediments (not involving active processes) can occur if sands as opposed to silt and clays are used [5], and applied at high deposition rates and administered to corals briefly over several minutes.

Where passive processes play a significant role is the interaction with the active mechanisms of ciliary transport. From the many time series observations of sediment smothering over the dredging period and based on laboratory-based studies with the same or similar species and morphologies, it seems that sediment shedding ability (and hence tolerance), is simply related to the opportunity for sediments to be moved in an uninterrupted downward direction. Sediments will ultimately be shed from the colony when an edge or the base of the colony is encountered. Several earlier studies have suggested that there is typically no co-ordinated movement or ‘routing’ of sediments to the edges of corals for shedding, and our observations largely support this. The sediment movement process across corals has been described as a random walk [43]. However, the images collected during the dredging project clearly show that sediment transport is highly dependent on surface orientation. Experimentally it has also been shown that sediments are shed faster over descending slopes with a rate related to the angle [41, 44, 45]. By corollary, transport is slower on upward slopes. We suggest that sediment shedding in corals is via ciliary movement and mucociliary transport and is an active process and an indiscriminate random walk, but sediment transport will inevitably be quicker on downhill slopes and quicker under increased flow [38, 42]. If the transport process encounters an edge the sediments will be shed or if encountering an upward slope, it is slowed (or potentially stops if the slope is very steep) and continued sediment deposition will cause sediments to pool. Further deposition on top of uncleared sediment deepens and enlarges the pool and results in some of the ‘snow-covered’, smothered images seen in this study. If trapped in local minima and with no downhill pathway for shedding, such pools of sediment require substantial wave action to be removed through re-suspension

Sediment re-suspension from the corals’ surface was seen in the time series on several occasions where deposits were eroded from the corals’ surfaces by waves, allowing an inspection of the fate of the underlying tissues. Previously smothered areas frequently showed bleaching (discolouration), especially in the massive *Porites* spp. and *Turbinaria* spp. Similar bleaching has also been induced by experimental smothering of *Porites* spp. corals and *Montipora aequituberculata* with sediment in aquarium-based experiments [42]. The bleaching *in vitro* was reversible and corals began regaining their colouration over a few weeks if the sediment was removed [42]. Similar re-colouration of bleached tissues, and over a similar time course, was also observed *in situ* during the dredging program. Bleaching in these instances is likely to be caused by either the complete loss of light that occurs under the layers of fine (silt and clay sized) sediments, but not coarse sands, although reduced solute exchange and anoxia cannot be discounted [5, 12].

Different species and morphologies possess different mechanisms to cope with higher levels of sediment deposition. Despite experiencing high levels of smothering and damage some encrusting morphologies were nevertheless quite capable of growing and extending at the distal, leading edges during the dredging program. Similarly, funnel shaped *Turbinaria* spp. often occur naturally with central lesions, referred to as ‘sacrificial sediment traps’ [46]. This was seen in this study by the formation of the central, basal lesion in the *Turbinaria* spp. which occurred even before the dredging started. Growth and reparation of the central lesion was observed in such colonies during the dredging phase, when the surface was temporarily sediment-free. Similar patterns of repair were also seen in massive *Porites* spp. during periods when the surfaces were sediment-free in between successive bouts of deposition and smothering.

The production of mucous sheets represents another mechanism to cope with higher levels of sediment in the massive *Porites* spp. These form when sediment loads become too high and subsequent sloughing of the sediment-impregnated mucous sheets effectively resets the surface to a sediment-free one. The response has been likened to the human sneeze reflex [31]. The massive *Porites* spp. are prominent components of reef assemblages in the Indo-Pacific region, and significant frame-work builders[30]. Mucous sheet formation and the ability to episodically reset their surfaces to a clean state is very likely to be one of the reasons why they can live in turbid inshore areas, despite a seemingly poor ability to rid themselves of settling and settled sediments [11]. Mucous sheet formation was also observed in branching *Porites* spp. and use of mucus was frequently seen in some *Turbinaria* spp. However, for the *Turbinaria* spp. the mucus was in the form of mesh like webs or filaments which rolled around the surfaces and collected sediments until shed. Similar use of mucus in *Turbinaria mesenterina* has been seen in laboratory experiments (see Fig 2 in [47]).

The differential effects of sediment smothering on the various morphologies examined indicates a well known trade-off. Corals with angled surfaces are very adept at shifting sediments and preventing smothering, but at the cost of light harvesting. Morphologies with flatter planes of upwards facing surfaces are better suited to light capture but at the cost of greater susceptibility to smothering. Smothering susceptible morphologies appear to have other adaptations to cope, including the ability to grow at the distal edges despite proximal smothering, to tolerate and repair lesions caused by smothering during clement, sediment-free periods, and for some taxa to use mucus and mucous sheet formation to episodically reset their surfaces.

### Management implications

Understanding the size of a deposition zone and the area within that deposition where smothering of corals occurs is fundamentally important for defining zones of high impact, for managing dredging using zonation schemes and for impact prediction at the EIA stage. Corals normally keep their surfaces sediment-free, and simple assessments of the percentage of the surface covered by sediment (or mucus) as used in this study is a very practical and rapid survey technique for monitoring and could be conducted by roving diver techniques or even diver-less assessments using remotely operated vehicles. Given the clear association of morality with smothering and the sediment deposition field, the technique could be used to confirm model predictions of sediment transport and fate and the zones of high and medium impact.

Sedimentation is usually cited as one of a suite of different pressures that has caused a decline in many reefs linked to terrestrial runoff and changed land-use practices. However, sedimentation rates have never really been measured on reefs at the appropriate scales [5, 16, 18, 19, 48], but see [22]. Information from sediments traps do not provide information on sedimentation rates in energetic environments, and misinterpretation and misuse of the information could have led to a misunderstanding of coral reef processes [16]. Whilst clearly a key-cause effect pathway for the impact of dredging where sediments are released into low energy water columns where deposition rates will be high, notions of sedimentation stress and damage under terrestrial runoff and natural resuspension events seem incompatible with the sediment-shifting ability shown by many of the corals in this study. This is especially so for the branching morphologies which tolerated extreme levels of sediment deposition. Perhaps for juvenile corals sedimentation poses more of a risk [49, 50], but perhaps also the term sedimentation is being used too generally to refer to sediment loading and cause-effect pathways associated with turbidity and changes in light quality and quantity, rather than sedimentation *per se*.
